# The role of reagent adding sequence in the NH_4_^+^-N recovery by MAP method

**DOI:** 10.1038/s41598-020-64634-9

**Published:** 2020-05-06

**Authors:** Caiqing He, Yunnen Chen, Chen Liu, Yang Jiang, Ruoyu Yin, Tingsheng Qiu

**Affiliations:** Jiangxi Key Laboratory of Mining & Metallurgy Environmental Pollution Control, Jiangxi University of Science and Technology, Ganzhou, 341000 PR China

**Keywords:** Environmental sciences, Environmental social sciences, Health care

## Abstract

Ammonium-nitrogen (NH_4_^+^-N) recovery from high concentration of NH_4_^+^-N-containing wastewater by struvite (MgNH_4_PO_4_·6H_2_O, MAP) precipitation method has been realized, but whether NH_4_^+^-N recovery under different reagent adding sequence of NaOH, solid Mg salt and P salt can generate different effects, remains ambiguous. In view of the problem, four modes to add reagents were investigated in detail on the formation of struvite. The results show that the Mode IV (M-IV, i.e. using 50% NH_4_^+^-N wastewater to dissolve completely the Mg salt and the P salt, respectively and then simultaneously poured into a beaker to mix the solution evenly and adjust the pH to 9.5.) has the highest NH_4_^+^-N recovery efficiency (90.80%) and the maximum mass of precipitates (896 mg) because of the more amount of alkali and initial seed formation. From the morphology of the obtained precipitates, it can be seen that sample M-IV is more loose and porous than the others. XRD patterns show that the four products under the different modes basically agree with the standard MAP.

## Introduction

Water environment deterioration and eutrophication of natural water bodies such as rivers, lakes and wetlands in China have been caused wide public concern in the last few decades^1^. High concentration NH_4_^+^-N is commonly present in textile, fertilizer, petrochemical, pharmaceutical industrial wastewater^2–5^. Meanwhile, nitrogen (N) is one of the indispensable nutrients for crops^6,7^. Therefore, it is necessary to recover NH_4_^+^-N from wastewater containing NH_4_^+^-N. Several methods have been applied to pretreat wastewater containing high concentration NH_4_^+^-N, such as steam-stripping / air-stripping means^8^, biological denitrification technology^9,10^ and MAP method. Struvite precipitation method^11–14^ is considered to be an attractive treatment option due to its simple operating, high efficiency, low cost, and can recover NH_4_^+^-N as slow-release fertilizer^15–17^, but different reaction conditions directly affect the recovery efficiency of struvite.

Many researchers have been working to improve the reaction conditions of struvite precipitation, include the solution of pH^18^, the molar ratio of Mg, P, N^3^ and combined struvite precipitation and microwave irradiation^19^. To the best of my knowledge, the sequence of adding reagents is an important aspect of the mineral flotation influencing factors studied by Zhang Qidong^20^ and Wang Jizhen^21^. But there have been few reports on the effect of the order of reagent addition on NH_4_^+^-N recovery and removal. In addition, in practical applications, the reagent addition sequence introduced in this article is mainly applicable to actual wastewater with little or no Mg salt and P salt, such as coking wastewater^19^ and hydrometallurgy wastewater^22^.

Based on the aforementioned information, the effect of the reagent addition sequence of solution on the formation of struvite was investigated. The corresponding precipitation products were characterized by scanning electron microscopy (SEM) and X-ray diffraction (XRD).

## Results and discussion

### The effect of reagent addition sequence on NH4^+^-N recovery efficiency

The NH_4_^+^-N concentration of solution, pH, and the molar ratio of Mg, P, and N were 500 mg L^−1^, 9.5, and 1.3:1:1, respectively. The reaction mechanism of struvite formation can be expressed as the following formula:

1 $${{\rm{N}}{\rm{H}}}_{4}^{+}+{{\rm{M}}{\rm{g}}}^{2+}+{{\rm{H}}{\rm{P}}{\rm{O}}}_{4}^{2-}+6{{\rm{H}}}_{2}{\rm{O}}\to {{\rm{M}}{\rm{g}}{\rm{N}}{\rm{H}}}_{4}{{\rm{P}}{\rm{O}}}_{4}\cdot 6{{\rm{H}}}_{2}{\rm{O}}\downarrow +{{\rm{H}}}^{+}$$

**Figure 1:**
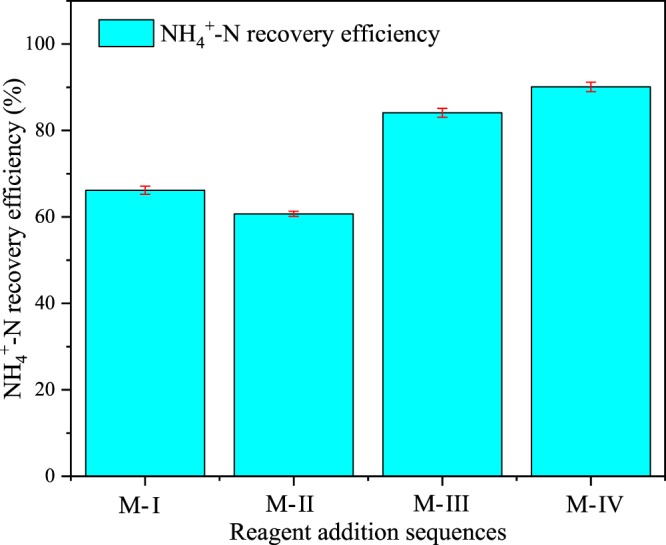
NH_4_^+^-N recovery efficiency (%) under four modes.

It can be seen from Fig. 1 that the sequence of reagent addition has a great influence on the NH_4_^+^-N recovery efficiency. Among the four modes, the M-IV has the highest NH_4_^+^-N recovery efficiency, up to 90.80%. The NH_4_^+^-N recovery efficiency in the other three modes is 66.17%, 60.70%, and 84.08%, respectively.

The significant difference in the recovery efficiency of NH_4_^+^-N may be related to the sequence of pH adjustment of the solution. As elucidated by Eq. (1), when the Mg salt and the P salt reacted with the NH_4_^+^-N wastewater to form struvite crystallization, the pH of solution will decrease. It is reflected in the Fig. 1 that when the pH was adjusted after reagent reaction (M-III and M-IV), the NH_4_^+^-N recovery rate were larger than that of pH value adjusted before the reaction (M-I and M-II). When the NaOH solution was used to adjust the pH to 9.5, it was confirmed that the latter two (M-III, 2.4 ml and M-IV, 2.4 ml) used more alkaline solution than the first two (M-I, 1.3 ml and M-II, 1.2 ml). This is a powerful explanation of why the latter two (M-III and M-IV) are significantly better than the former two (M-I and M-II) for NH_4_^+^-N recovery rate. Consequently, one of the mechanisms by which different reagent addition sequences lead to different ammonia nitrogen recovery rates can be attributed to the discrepancy in the final NaOH addition amount. Additionally, other possible causes will be explained in the next section on the effect of reagent addition sequence on precipitates mass recovery.

### The effect of reagent addition sequence on mass of precipitates

**Figure 2:**
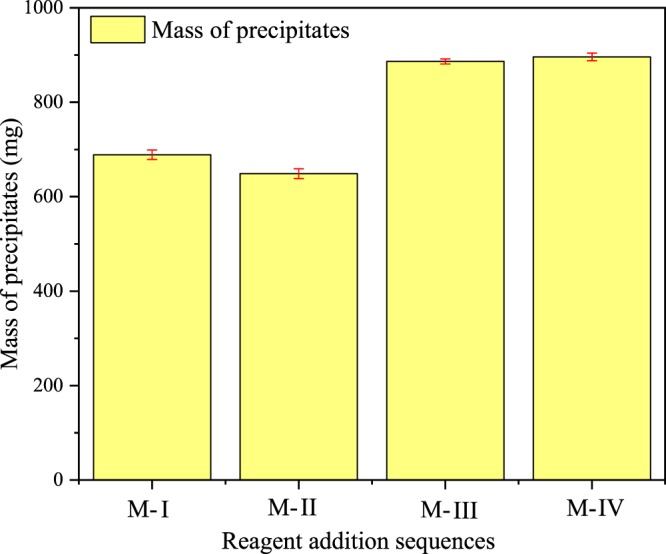
Mass of precipitates (mg) under four modes.

From Fig. 2 can be seen that the sequence of reagent addition has also great influence on the yield of precipitates. Among the four modes, the M-IV has the highest mass of sediment, up to 896 mg. The amount of sediment in the other three modes is 689 mg (M-I), 649 mg (M-II), and 886 mg (M-III), respectively. Compared with Fig. 1, it can also be observed that the higher NH_4_^+^-N recovery efficiency, the more precipitates are produced.

Meanwhile, the reason why the M-IV is better than the M-III is probably that the difference in seed amount of crystals formation, which is conducive to the increase of precipitation^23^. For M-IV, it was ensured that Mg salt and P salt solids were dissolved in NH_4_^+^-N wastewater, respectively, before adjusting the pH to 9.5, then the three reagents reacted with each other and formed the initial crystal seed^24^. By contrast, P salts took longer (30 seconds–1 minute) to dissolve completely than Mg salts when Mg salt and P salt reagents added into NH_4_^+^-N wastewater, which affected the formation of crystal seed, leading to a bit lower mass of precipitates in M-III than that of M-IV^25^.

### SEM analyses

**Figure 3:**
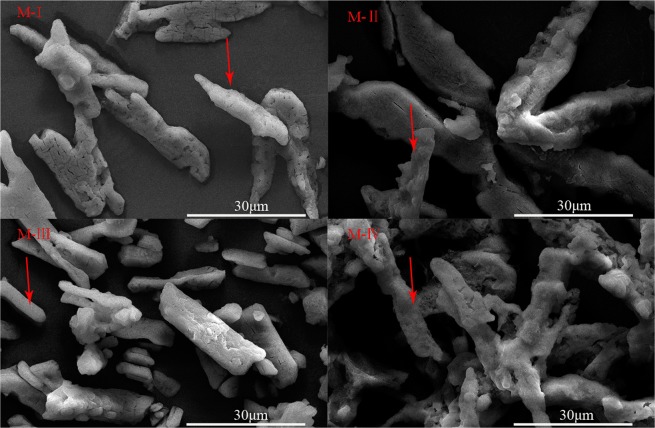
SEM images of products under different reagent addition sequences.

Figure 3 shows the SEM images of products obtained under different reagent addition sequences. According to the results shown in Fig. 3, the morphology of products formed in four modes was quite different. In particular, the orthorhombic crystal phases of M-II and M-IV differ greatly, in which M-II is broad and dense and M-IV thin and loose. This is because in order to promote the formation of precipitation, the compaction degree of solids is affected, thus becoming loose and difficult to further precipitate. This result is similar to Song *et al*.^26^. This also in accord with the results analyzed in Fig. 1. The amount of sodium hydroxide has an effect on the morphology.

### X-ray diffraction

**Figure 4:**
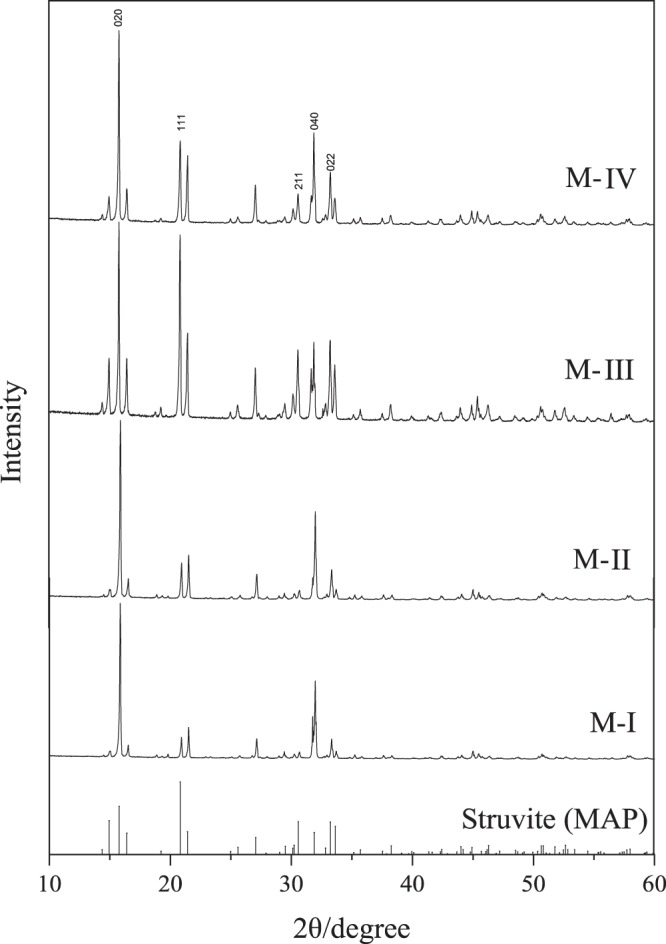
The XRD spectra of products under different reagent addition sequences.

Figure 4 shows the XRD spectra of products obtained under different reagent addition sequences. As can be seen from the XRD diffractogram (Fig. 4), the (020), (111) and (040) reflections of the four spectra are almost identical with the standard struvite spectrum, which can be confirmed that struvite crystal is the mainly component in the obtained precipitates according to JCPDS files (15–0762). For the peak’s intensity of (022) and (211) reflection, M-III and M-IV were stronger than that of M-I and M-II. This is due to the more sodium hydroxide retained by M-III and M-IV, which is consistent with the results obtained in Fig. 1.

## Conclusions

The experimental results showed that the sequence of reagent addition has a great influence on NH_4_^+^-N recovery and mass of precipitates. The optimal NH_4_^+^-N recovery efficiency (90.80%) appeared in M-IV, that is, using 50% NH_4_^+^-N wastewater to dissolve completely the Mg salt and the P salt, respectively and then simultaneously poured into a beaker to mix the solution evenly and adjust the pH to 9.5. The mechanism for M-IV mainly resulted from the more alkali than M-I and M-II, and the more amount of initially crystal seed formation than M-III.

## Materials and Methods

### Materials

All reagents were of analytical grade and purchased in Shantou Xiqiao Science Co., Ltd., Guangdong, China. P salt and Mg salt used in the experiment were disodium hydrogen phosphate dodecahydrate (Na_2_HPO_4_·12H_2_O) and magnesium chloride hexahydrate (MgCl_2_·6H_2_O), respectively. Ammonium chloride (NH_4_Cl) was used to prepare simulated NH_4_^+^-N wastewater. The initial pH of solution was adjusted with 1 mol L^−1^ NaOH and HCl. All solvents were deionized water.

### Procedures of experiments

**Figure 5:**
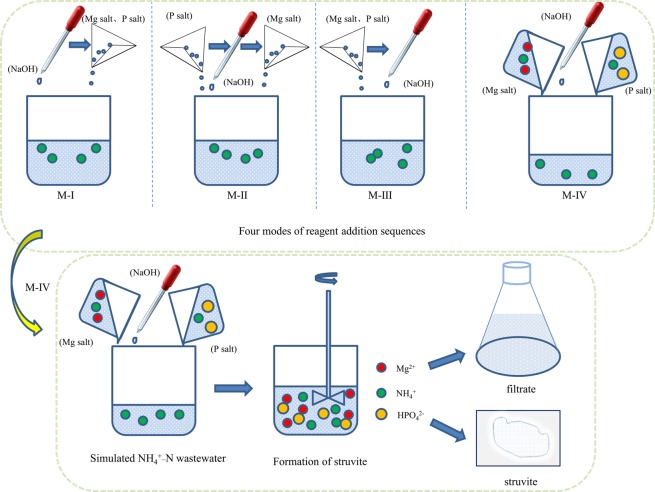
Four modes of reagent addition sequences schematic diagram of struvite formation.

Under room temperature (25 ± 2°C), four modes of reagent addition sequences are as follows: Three 200 ml beakers were filled with 100 ml 500 mg L^−1^ synthetic NH_4_^+^-N wastewater. In the beaker I (M-I), the initial pH of solution was adjusted to 9.5 firstly, then added solid Mg salt and P salt at the same time and stirred to dissolve completely. In the beaker II (M-II), solid P salt was added and stirred to dissolve completely, then adjusted the pH of the mixture to 9.5, following Mg salt was added and stirred to dissolve completely. For the beaker III (M-III), Mg salt and P salt were added at the same time and then stirred to dissolve completely, following adjusted the pH of the solution to 9.5. As for the beaker IV (M-IV), Mg salt and P salt were first added into a 100 ml beaker containing 50 mL of NH_4_^+^-N wastewater and then stirred to dissolve completely, respectively. Next simultaneously poured into a 200 ml beaker to mix the solution evenly and adjust the pH to 9.5. Four modes of reagent addition sequences and schematic diagram of struvite formation have shown as Fig. 5.

The mixed solution of the four beakers was stirred by a magnetic stirrer to promote the formation of precipitates and then standing for 30 min to make the precipitation complete. After that, precipitation yield was obtained after filtrating through a 0.45-μm filter membrane, washing by deionized water three times and drying in an oven at 50°C. The content of NH_4_^+^-N in the filtrate was determined. All batch experiments were performed in triplicate and the average was taken as the experimental results.

### Analytical methods

The morphology of the precipitates was characterized using a scanning electron microscope (SEM, MLA650F, FEI, Hillsboro, USA). The crystal patterns of the synthesized struvite was obtained by X-ray diffractometer with Cu-Kα radiation (40KV, 40 mA), from radiation angle 10–60°(XRD, DX-2700, Shjingmi, Shanghai, China). The content of NH_4_^+^-N in solution was measured by visible spectrophotometer (722 N, INESA, Shanghai, China) with a wavelength of 420 nm according to Nessler’s reagent spectrophotometry method.

The calculation Eq. (2) for the recovery of NH_4_^+^-N is:

2 $$Y=\frac{{C}_{O}-{C}_{t}}{{C}_{O}}\times 100$$

where *Y* (%) is the recovery efficiency of NH_4_^+^-N, *C*_0_ refers to the initial NH_4_^+^-N concentration (mg L^−1^) and *C*_*t*_ refers to the equilibrium NH_4_^+^-N concentration (mg L^−1^) of the solution.
